# Immune Evasion Strategies of Schistosomes

**DOI:** 10.3389/fimmu.2020.624178

**Published:** 2021-02-04

**Authors:** Jacob R. Hambrook, Patrick C. Hanington

**Affiliations:** School of Public Health, University of Alberta, Edmonton, AB, Canada

**Keywords:** schistosome, schistosomiasis, immune evasion, immunomodulation, *Biomphalaria glabrata*

## Abstract

Human schistosomes combat the unique immune systems of two vastly different hosts during their indirect life cycles. In gastropod molluscs, they face a potent innate immune response composed of variable immune recognition molecules and highly phagocytic hemocytes. In humans, a wide variety of innate and adaptive immune processes exist in proximity to these parasites throughout their lifespan. To survive and thrive as the second most common parasitic disease in humans, schistosomes have evolved many techniques to avoid and combat these targeted host responses. Among these techniques are molecular mimicry of host antigens, the utilization of an immune resistant outer tegument, the secretion of several potent proteases, and targeted release of specific immunomodulatory factors affecting immune cell functions. This review seeks to describe these key immune evasion mechanisms, among others, which schistosomes use to survive in both of their hosts. After diving into foundational observational studies of the processes mediating the establishment of schistosome infections, more recent transcriptomic and proteomic studies revealing crucial components of the host/parasite molecular interface are discussed. In order to combat this debilitating and lethal disease, a comprehensive understanding of schistosome immune evasion strategies is necessary for the development of novel therapeutics and treatment plans, necessitating the discussion of the numerous ways in which these parasitic flatworms overcome the immune responses of both hosts.

## Introduction to Schistosomes/Immunosuppression

All organisms must deal with threats in their environments to survive and thrive. The same is true of parasites, whose environments put them constantly at odds with host immune systems. Whereas some parasites have only one host and must therefore develop immune evasion/suppression tactics against only one type of immune system, others employ indirect life cycles, which means they encounter diverse immune systems that utilize unique strategies geared towards their death and destruction. Schistosomes, which are digenean trematodes of the genus *Schistosoma*, are one such parasite. These flatworms employ an indirect life cycle, alternating between a gastropod mollusc as an obligate intermediate host and a definitive vertebrate host such as a human (1). Schistosomiasis, the disease caused by schistosome infection of humans, is widely considered the second most important parasitic disease from a public health perspective, trailing only malaria. It afflicts an estimated 206 million people, killing up to 200,000 annually, and resulting in an estimated loss of between 1.9 and 3.3 million disability adjusted life years (2–4). Thus, a comprehensive examination and understanding of how these parasites evade the immune systems of such drastically different hosts has been and continues to be the focus of extensive investigation (5–7). This review seeks to highlight the foundational work which demonstrated schistosome evasion/suppression of the immune response in both host systems. Those specific components that have been implicated as being immunomodulatory, and other uncharacterized factors that bear similarities to known parasite immunomodulatory factors will be discussed.

### The Gastropod Immune System

**Figure 1:**
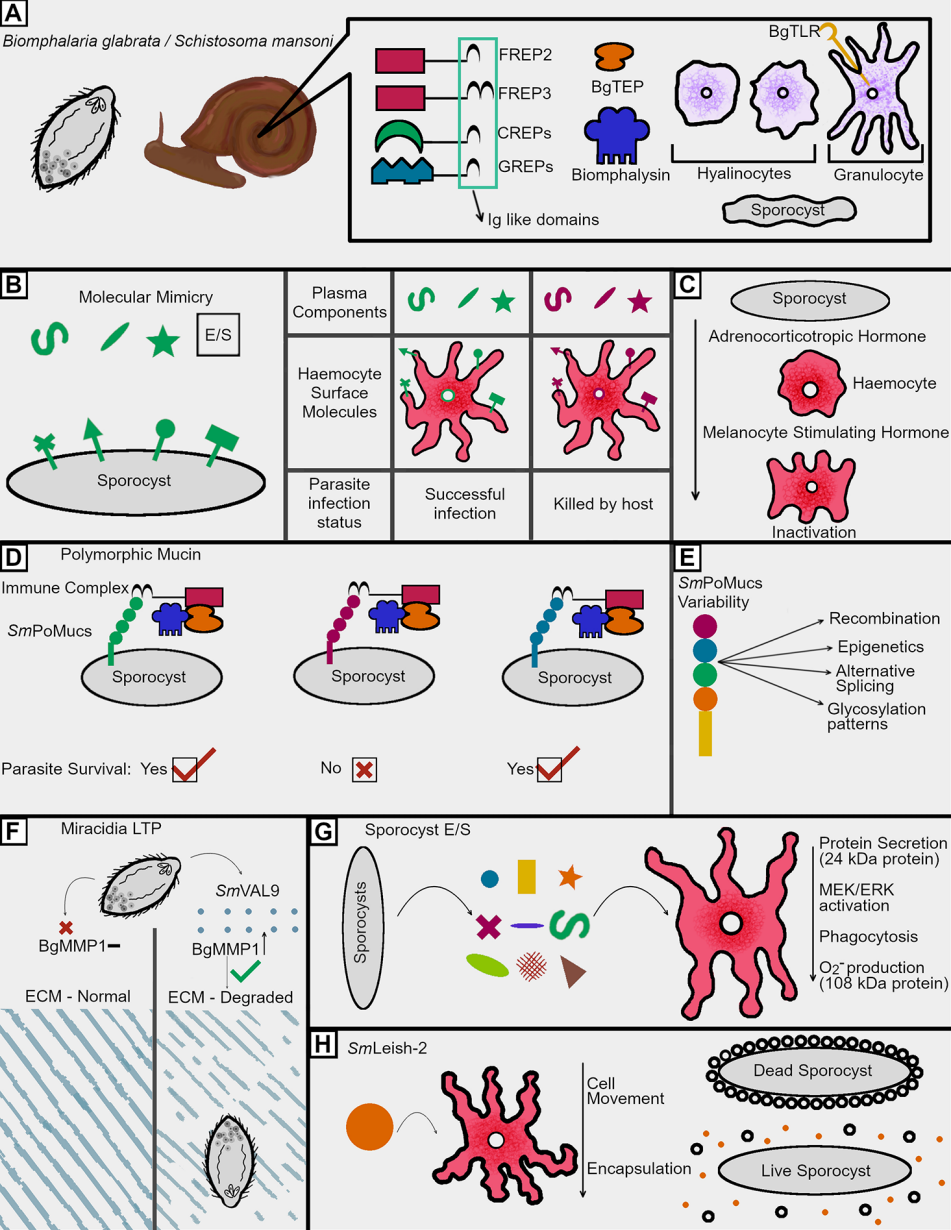
Immunosuppression tactics in the intermediate host. **(A)** Snails employ a wide variety of both humoral and cellular factors in combatting schistosome infections, many of which incorporate with each other to facilitate parasite killing. The humoral factors are largely composed of PRRs but also feature cytotoxic components such as biomphalysin. The cellular arm of the immune response features *Bg*TLR displaying granulocytes, which envelope invading schistosomes, and hyalinocytes, which seemingly focus on the production of humoral factors and cell signaling molecules. **(B)** Parasites employ molecular mimicry by utilizing surface molecules and E/S products which share glycosylation patterns seen in snail plasma and on the surface of circulating hemocytes. Sharing such glycosylation patterns has been shown to correlate with survival during infection, suggesting these shared epitopes help schistosomes avoid recognition within the snail. **(C)** Molecular mimicry is also employed by the production of immune cell inactivating hormones like those produced by the snail which renders normally lethal hemocytes inactive and unable to kill invading sporocysts. **(D)** In order to avoid recognition by host pattern recognition receptors, schistosomes employ a highly variable series of polymorphic mucins. These mucins are recognized by host *Bg*FREPs, and the variable nature of both the mucins and FREPs has led to the understanding that successful recognition of *Sm*PoMucs by FREPs is a key determinant of infection success. If the host FREP can recognize the *Sm*PoMucs and the surface of a sporocyst, that sporocyst will likely be killed, while having an unrecognizable *Sm*PoMuc leads to immune evasion by avoiding *Bg*FREP recognition. Such killing is thought to be at least partially dependent on a humoral *Bg*FREP/*Bg*TEP/Biomphalysin complex. **(E)** In order to generate a highly variable surface mucin, numerous processes occur to give rise to the considerable amount of variability seen between different sporocyst *Sm*PoMucs. **(F)** Miracidia employ a venom allergen like protein which has been shown to upregulate the production of a *B. glabrata* matrix metalloproteinase (*Bg*MMP1). This metalloproteinase is hypothesized to facilitate degradation of host connective tissues. Such degradation would allow for easier movement further into the host during initial infection. **(G)** E/S products from developing sporocysts facilitate the downregulation of key anti-parasitic functions in hemocytes. While some of these proteins have been identified, others remain of an unknown composition and are merely referred to by their size. **(H)** SmLeish-2 released by the parasite reduces hemocyte motility and therefore downregulates parasite encapsulation. This allows for continued movement and development of the sporocyst within the host.

After emerging from its egg into freshwater, the miracidial stage of the schistosome life cycle seeks out a suitable snail host. Although the immune systems of snails lack the overall complexity and antibody mediated adaptive response seen in mammals, these invertebrates combat schistosome infections using a multitude of immune strategies resulting in larval damage and ultimately the killing of invading schistosomes (**Figure 1A**) (8, 9). Research into the immunological underpinnings of snail/schistosome interactions has been undertaken largely using the *Schistosoma mansoni/Biomphalaria glabrata* model system that exploits numerous *B. glabrata* strains that display different compatibility profiles with specific strains of *S. mansoni*. This allows for detailed examination of both the host immune response as well as parasite survival strategies (10, 11).

Killing and elimination of invading miracidia and the sporocysts that they transform into relies primarily on coordination between the cellular and humoral immune responses of the snail. The immune cells, termed hemocytes, locate, surround, and encapsulate invading parasites. *B. glabrata* also features numerous immune molecules hypothesized to function in recognition of invading schistosomes such as the wide variety of leucine rich repeat-containing receptors including some canonical toll-like receptors (TLRs) (**Figure 1A**) and other pattern recognition receptors (PRRs) such as peptidoglycan recognition receptors (PGRPs), variable immunoglobulin and lectin domain-containing molecules, and the proteins encoded for in the Guadeloupe resistance complex (GRC) (8, 12–15).

As mentioned above, the humoral branch of the *B. glabrata* immune system is characterized as being important for recognition and clearance of *S. mansoni* and is largely composed of pattern recognition receptors (PRRs). Many of these PRRs have been found to contain at least one immunoglobulin superfamily (IgSF) domain next to an interceding region (ICR) (**Figure 1A**). Some of these ICRs are linked to galectin like domains, while others are linked to C-type lectin domains. These PRRs are referred to as Galectin-related proteins (GREPs) and C-type lectin related proteins (CREPS) (12).The most well characterized PRR in *B. glabrata* are the Fibrinogen related proteins (FREPs), which feature one or two IgSF domains linked to a fibrinogen like domain by an ICR (14). At least one FREP, FREP3, has the capacity to bind the surface of sporocysts and act as a opsonin, while siRNA mediated knockdown of FREP3 reduces resistance to *S. mansoni* challenge (16). *B. glabrata* FREPs (*Bg*FREPs) possess the capacity to somatically diversify, which is a unique among invertebrates (17). Given the observation that schistosomes are in possession of polymorphic surface mucins that interact with FREPs, this somatic diversification suggests a possible form of adaptive immunity. This potential adaptive immunity is the subject of numerous investigations (18–20). In addition to these immunoglobulin-like domain containing PRRs, *B. glabrata* also possesses several thioester containing proteins (TEPs), which resemble complement component C3 in both form and function (21). The humoral immune response is not merely relegated to pathogen recognition; a *β*-pore forming toxin (*β*-PFT) known as biomphalysin is also able to directly kill *S. mansoni* sporocysts (22). Rather than functioning independently, at least three humoral immune components (*Bg*FREPS, *Bg*TEP, and biomphalysin) function as a unit to target and facilitate elimination of developing sporocysts (**Figure 1D**) (18).

### Molecular Mimicry in the Gastropod Host

Avoiding immune recognition and destruction is mediated by a wide array of immune evasion techniques employed by invading miracidia and developing sporocysts (**Figure 1B**). First referred to as molecular mimicry in 1964, the process of parasites displaying antigens similar to those of their hosts has been a research topic of interest in numerous pathogen model systems (23, 24). The phenomenon has been shown to span numerous parasitological phyla, with some helminths utilizing glycans to mask their presence to their hosts (25). Some parasitic nematodes even employ glycans typically only found in vertebrates, suggesting that their use in parasites acts to hide the pathogen from the immune response (26).

The study of molecular mimicry in intermediate hosts was first reported in 1965 when in was observed that *B. glabrata* possessed similar antigens to those found in developing *Schistosoma mansoni* larvae (**Figure 1B**) (27). This was later confirmed by the development of polyclonal antibodies to hemolymph from *S. mansoni* resistant (10-R2) and susceptible (M-line) *B. glabrata* strains, with both reacting strongly with the surface of *S. mansoni* miracidia and sporocysts (28, 29). This association persisted for at least 48 h post transformation from miracidia to sporocyst, suggesting the developing larvae consistently share at least certain surface proteins during the first 48 h of infection, during which, they are most likely to be targeted by the snail immune response (28). Similarly, antibodies raised against whole sporocysts interact with the surface of *B. glabrata* hemocytes (30). Although these cross-reactive immunoglobulin tests suggest some form of molecular mimicry, such studies lack the necessary specificity for examination of shared antigens. Observed cross reactivity may simply be due to shared glycosylation patterns between the two invertebrates, with a variety of carbohydrates epitopes being found in both animals (31, 32). Often, such shared glycosylation patterns are featured at the surface of miracidia and sporocysts, although they may also be found in E/S products (33, 34). Although such similarities have suggested the employment of molecular mimicry for several decades, potential targets for such a phenomenon have emerged more recently. Differences in N-glycosylation patterns of hemolymph in the resistant BS-90 strain *B. glabrata* featured less glycan epitope similarities to *S. mansoni* than a susceptible *B. glabrata* strain (*Bg*PR). This data provides support for the hypothesis that molecular mimicry might aid *S. mansoni* to avoid recognition by the varying lectins employed by *B. glabrata* as a means of immune recognition (35). This is further supported by the observation that hemolymph from susceptible *B. glabrata* strains, such as the NMRI snail strain, features a greater abundance of schistosome-like glycan epitopes in their hemolymph than resistant BS-90 strain snails (**Figure 1B**). Larval transformation products (LTPs) released by the parasite during miracidium-to-sporocyst transformation also participate in glycan mimicry (**Figure 1B**). Host hemolymph proteins and circulating hemocytes are capable of reacting with LTPs (36, 37). A differential binding pattern between LTPs and hemolyph proteins isolated from *B. glabrata* strains of varying compatibility with *S. mansoni* is observed using far-western blotting. This suggests the exploitation of differing glycan epitopes by *S. mansoni* during larval transformation (37). The attempts at mimicry made by *S. mansoni* are not limited to surface level epitopes, as sporocysts possess the capacity to produce host-like adrenocorticotropic hormone, which is processed by host hemocytes to become melanocyte-stimulating hormone, resulting in the rounding of hemocytes near the sporocyst (**Figure 1C**) (38).

### Polymorphic Mucins

While molecular mimicry may very well function in hiding the parasite from the immune response of the snail, a new set of molecules that are distinct to the parasite have emerged as possible determinants of survival: *S. mansoni* polymorphic mucins or *Sm*PoMucs (**Figure 1D**). These diverse proteins were first identified as part of a proteomics screen seeking to identify differentially abundant proteins produced by *B. glabrata-*compatible and incompatible strains of *S. mansoni*, and have gone on to be one of the most intensely studied components of resistance polymorphism in the *B. glabrata/S. mansoni* system (20, 39, 40). *Sm*PoMucs consist of three distinct groups, each containing a characteristic C-terminal domain attached to a variable number of tandem repeats ranging from n = 1 to n~55 (41). These repeats, heavily composed of serine, threonine, and proline residues, allow for heavy glycosylation of these proteins, aiding in their classification as mucin-like molecules (41). Through a series of events including recombination, expression polymorphism, inheritable acetylation-based epigenetics, various mechanisms of post translational splicing, and differential glycosylation patterns, *S. mansoni* is capable of generating numerous different polymorphisms while only being in possession of an estimated 10 *Sm*PoMuc genes (**Figure 1E**) (42–44). While different *S. mansoni* strains vary in their *Sm*PoMucs transcript sequences and expression patterns of, such differences are also seen on an individual level between sporocysts (42, 43). In 2010, Moné and associates made the key observation that these diverse molecules could be found in association with somatically diversifying host immune molecules; FREPs and *Bg*TEP (19). This is significant seeing as research demonstrating that a *Bg*FREP/*Bg*TEP complex can render susceptible snail hemocytes and plasma nearly as deadly to *S. mansoni* sporocysts as plasma from *S. mansoni-*resistant *B. glabrata* strains. Given the fact that *Sm*PoMucs are found associated with the apical glands and E/S products of miracidia and sporocysts, *Sm*PoMucs variability should be considered a key mechanism by which the parasite discourages recognition by key humoral immune complexes both at the surface of the parasite and in the surrounding tissues (18, 19, 40, 41). Given the somatic variation in *Bg*FREPs, and the highly polymorphic nature of *Sm*PoMucs, these interactions may prove to be supportive of the Red Queen Hypothesis whereby host and pathogen vary their respective molecular determinants of infection in order to survive (**Figure 1D**) (40).

### Miracidia

When penetrating a suitable snail host, schistosomes are not restricted to molecular mimicry to evade detection and attack from molluscan immune cells. The first barrier miracidia face during intermediate host infection is passage through host tissues, a process thought to be facilitated by a thiol proteinase located in the lateral penetration glands of miracidia developing inside eggs (45). During initial penetration, larval transformation products (LTPs) released from the schistosome are also expected to aid in penetration while subsequently facilitating immune evasion (46). LTP contents have been examined using mass spectrometry, which revealed the presence of numerous factors involved in immune modulation, including but not limited to: proteases (a calpain, serine peptidase, leishmanolysin like protein), protease inhibitors (alpha-macroglobulin, cystatin B), ion binding proteins, antioxidant enzymes, and venom allergen like proteins, which are the most abundantly featured proteins present (47). One such venom allergen like protein (*Sm*VAL9) has been shown to be key in facilitating the upregulation of extracellular matrix remodeling genes during penetration, as evidenced by *in vitro* observations using the *B. glabrata* embryonic cell line, suggesting that it may also be involved in movement throughout host tissues (**Figure 1F**) (48).

### Developing Sporocysts

After miracidial penetration of the snail host, the developing sporocyst goes on to synthesize excreted/secreted (E/S) factors designed to facilitate survival and immune evasion (**Figure 1G**). Although nomenclature differs throughout the literature, for the purposes of this discussion, E/S products differ from LTPs in that they are produced during primary sporocyst culture, while LTPs are release during the miracidium-to-sporocyst transformation. These combinations of carbohydrates and proteins are predominantly produced 1 day post entry into the snail, implicating them as determinants of infection success in avoiding encapsulation by host hemocytes, a process which leads to damage of the parasite *via* ROS production, followed by death and elimination by phagocytosis of dead parasite material (49). Both microarray and serial analysis of gene expression techniques have been used to examine expression profiles in sporocysts, with a variety of proteases (cathepsin C, preprocathepsin L, hemoglobinase, and elastase) and antioxidants (Cu/Zn superoxide dismutase, peroxiredoxins 1 and 2, and glutathione peroxidase) having been identified as upregulated factors potentially responsible for some of the inhibitory effects seen in the host (50, 51).

The effects of excreted/secreted schistosome products have largely been investigated by examining the effect of whole E/S products on various biochemical and immune related functions in host hemocytes. They alter hemocyte metabolism, as evidenced by their capacity to modulate protein secretion from hemocytes in various *B. glabrata* strains (**Figure 1G**) (52). They also modulate cell signaling, as evidenced by the fact that direct exposure of susceptible *B. glabrata* to E/S products reduces both MEK and ERK phosphorylation, two hallmarks of phosphokinase based signaling (53). Seeing as MEK and ERK phosphorylation are cell signaling pathways used to facilitate granulin mediated hemocyte proliferation, this reduction in phosphorylation may also serve to decrease the availability of appropriate levels of cytotoxic hemocyte populations (13). Another downstream result of ERK signaling is the presence of heat shock proteins, such as HSP70. HSP70 abundance decreases are susceptible to snail hemocytes exposed to *S. mansoni* E/S products (53). Given the role of HSPs in the stress response and as damage-associated molecular patterns (DAMPs), this may also be a mechanism by which schistosomes persist in their intermediate hosts (54). Exposure of hemocytes to E/S products can impact parasite targeting/recognition and killing (**Figure 1G**). These products are capable of suppressing hemocyte chemotaxis while also reducing their capacity to phagocytize foreign particles (55–57). They also prevent the production of superoxide anions and nitric oxide, a key method by which hemocytes kill invading sporocysts (58, 59).

To date, four specific factors present in the LTPs produced by *S. mansoni* have been functionally characterized. Upon entry into the snail, *S. mansoni* releases venom allergen like protein 9 (*Sm*VAL-9), which results in the upregulation of a *B. glabrata* matrix metalloprotease (48). Given the role of such metalloproteases in remodeling tissue, it is hypothesized that this facilitates entry and penetration of the parasite into host tissue (**Figure 1F**). Two more immune modulators were identified as part of an examination into E/S product synthesis by sporocysts *in vitro* (49). The first is a polypeptide of roughly 24 kDa found to be capable of inhibiting protein synthesis by snail hemocytes, an effect that was seen in susceptible M-line snails, but not observed in the more resistant 10-R2 strain (58). The second is a molecule of 108 kDa in size, predicted to be composed of more than one 50 kDa domains. This 108 kDa protein was shown to be able to scavenge superoxide anions produced by phagocytosis-stimulated M-line *B. glabrata* hemocytes, thereby protecting the parasite from this toxic oxygen species (58). A fourth and more recently characterized protein was an invadolysin upregulated by *S. mansoni* 33.2-fold at 12 h post infection in *B. glabrata.* This matrix metalloprotease is similar to Leishmanolysin (GP63), the predominant immunomodulatory protein found on the surface of *Leishmania* sp (60). This invadolysin, termed *Sm*Leish, was found to be capable of reducing the motility of susceptible M-line *B. glabrata* hemocytes (**Figure 1H**). This function is essential in reducing the frequency at which sporocysts are encapsulated by hemocytes *in vitro*, and was also found to be crucial for survival within *B. glabrata* (55).

## Introduction to the Human Host

After leaving their snail intermediate host, cercaria move to the surface of the water column in the hopes of encountering a compatible mammalian host. For *S. mansoni* and *S. haematobium*, this means encountering a human or small rodent such as a mouse or rat (61). *S. japonicum*, on the other hand, is capable of infecting over 40 different mammalian species, although humans are one of the primary hosts (62). The environment inside of a human host differs drastically from that of a gastropod mollusc. While snails mount a capable and robust innate response, in humans, the innate and adaptive arms of a schistosomicidal immune response are brought to bear (63). Each life cycle stage that takes place within the mammalian host is presented with a unique set of immune challenges based upon the location in the host. The exact efficacy of these host immune processes remains an investigated manner. Early work demonstrated that mammalian effector cells such as neutrophils and eosinophils are capable of killing schistosomulae *in vitro*, especially in the presence of complement proteins and anti-schistosome immunoglobulins (63–65). Whether or not this occurs *in vivo* remains unascertained. Resistance to schistosome infection does vary between age groups, with children and teenagers featuring higher reinfection rates than adults, although it is not known for certain if this increase in resistance is age mediated or antibody based (66–69). It is clear that sterile immunity is not induced in humans after infections, but models suggest that the levels of protective immunity that are developed are likely caused by exposure to dead worm antigens (70). To counter the numerous challenges schistosomes face in their definitive hosts, these worms have developed several immune evasion mechanisms allowing for high infection loads and lifespans up to 37 years, indicating their capacity to survive and thrive despite the host immune response (71).

### Initial Penetration

The first immune barrier that schistosomes encounter is the skin. The skin functions as a barrier to all pathogens and helps prevent the entry of parasites, fungi, bacteria, and viruses. The components of the skin relevant to schistosome infections are the epidermis, the dermis, and the basement membrane separating the two. The epidermis is largely composed of keratinocytes, which secrete lipids to aid in the formation of a barrier, but specialized dendritic cells known as Langerhans cells are also present and are capable of taking up antigen and migrating into the lymph system. The basement membrane is a network of connective molecules composed largely of Collagen type IV and Collagen type VII (72–74).. On the inner side of the basement membrane, the dermis exists as a layer of skin featuring nerve endings, hair follicles, muscles, and both blood and lymph vessels (72, 74).

**Figure 2:**
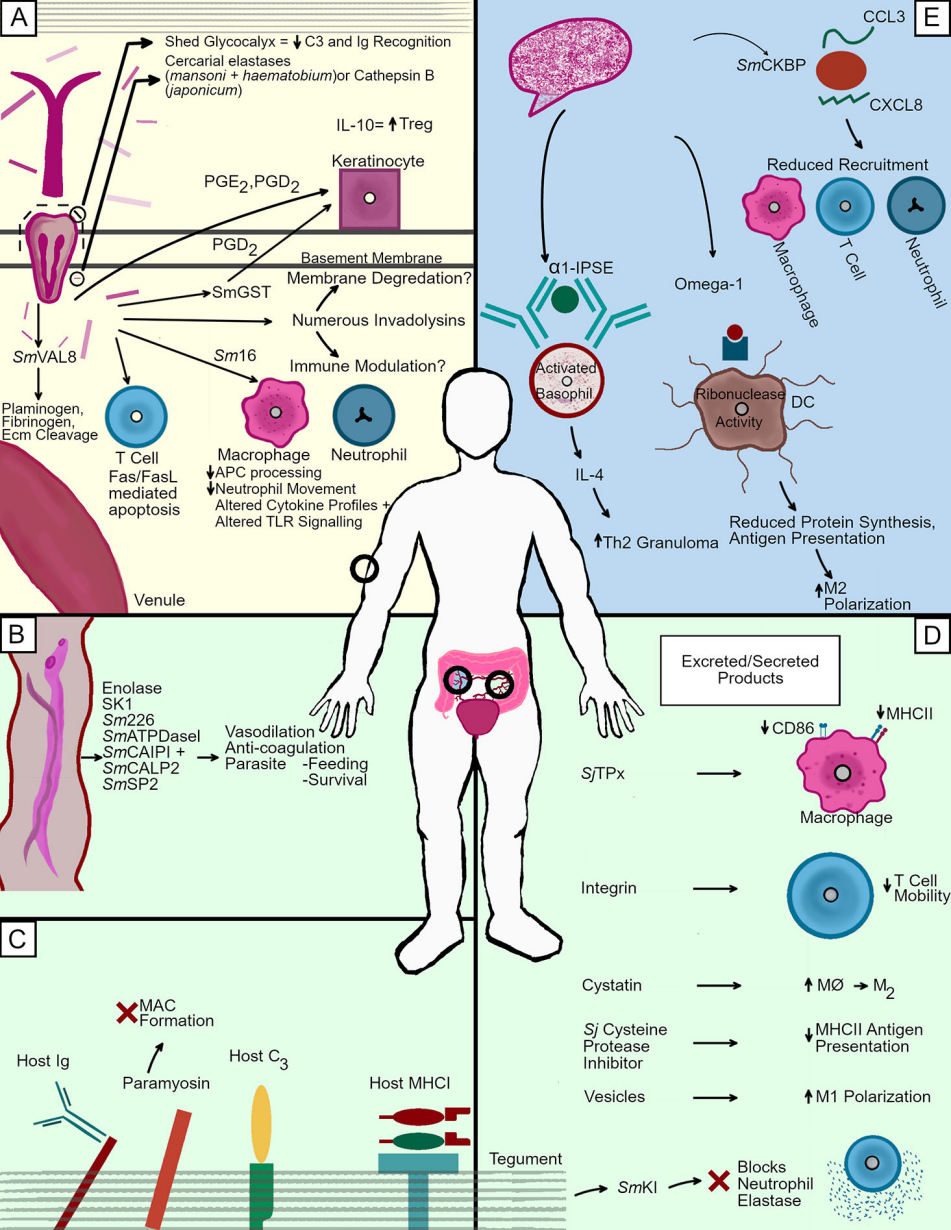
Immunosuppression tactics in the human host. Schistosomes travelling through employ different immune evasion methodologies depending upon their location and life cycle stage. **(A)** As cercariae penetrate the skin, numerous proteases of different families help facilitate the cleavage of host molecules. The degradation of key structural components such as numerous collagens and elastin by these proteases helps the parasite descend through the epidermis, penetrate through the basement membrane, and eventually navigate the dermis in the search for a nearby venule. The invading larvae also release numerous immunosuppressants such as Sm16 and prostaglandins which alter leukocyte function in an attempt to avoid cell mediated death by creating a favorable immune environment. **(B)** Adults can also release molecules to reduce blood clotting in the area immediately surrounding a mated pair. This wide variety of molecules may be involved in the degradation of host plasma components to facilitate feeding, but their capacity to act as anti coagulants also implies the creating of a milieu in which the worms are able to move freely without fear of being killed by the coagulation of the blood in which they are emersed. **(C)** Adult worms in the mesenteric blood vessels incorporate host molecules into their tegument. Many of these molecules are important immune factors, which are bound in such a way as to prevent proper opsonization. IgG is found *via* its Fc portion, making in unrecognizable to cell found Fc receptors. Complement component C3 is also bound, although the effects of this association are not completely understood. Finally host MHCI can also be found bound to adult worms *via* its *β*2-microglobulin domain. The worm also targets mammalian factors not only for capture, but also for inactivation, as evidenced by schistosome paramyosin inhibiting membrane attack complex formation. **(D)** Schistosomes release a wide variety of parasite derived factors into their environment to create tolerable conditions for their survival. These molecules perform many different tasks, including the inhibition of cysteine proteases (*Sj* cysteine protease inhibitor), the blocking of neutrophil elastase (*Sm*KI), and the alteration of antigen processing in macrophages (*Sj*Tpx). **(E)** Schistosome eggs release several factors which is key in skewing the T cell response towards the Th2 phenotype necessary for migration through the intestinal wall. These include *α*-1, which causes TH2 type granuloma formation through basophil mediated IL-4 production, and Ω-1, which brings about M2 polarization by altering dendritic cell antigen presentation. The chemokine binding protein *Sm*CKBP is also present and reduces recruitment of varying leukocytes.

In order to successfully develop as a schistosomula, cercariae must penetrate through the epidermis, basement membrane, and dermis in order to locate a venule or lymphatic vessel that will subsequently lead them to the lungs (75). A healthy debate exists as to the exact mechanism and kinetics by which this skin penetration occurs, but a variety of model systems have allowed for a better understanding of how cercariae complete this task. In mice, hamsters and rats, the time needed for half of *S. mansoni* to leave the skin is 88, 65, and 70 h, respectively (73). Studies using human skin explants have demonstrated that *S. mansoni* and *S. haematobium* seemingly have similar invasion kinetics to what is seen in rodent model systems, with penetration into the dermis and location of a venule taking between 48 and 72 h (76–78). Penetration by *S. japonicum* has been shown to proceed at a faster rate, with up to 90% of the parasites reaching the dermis/host venules within 24 h post infection, some even doing so in only 12 h (77). Such an rapid migration is also supported by the observation that *S. japonicum* reach the lungs of experimentally infected mice after 3 days, while *S. mansoni* and *S. haematobium* take 6 days (79). The time it takes for penetration to occur suggests that movement in the skin is not easy for the invading larvae, and suggests the need for developing mechanisms of avoid immune cells within both the epidermis and dermis, where numerous innate immune cells are present (**Figure 2A**).

Penetration through the uppermost layers of the epidermis is aided by the aqueous environment in which they encounter their host, seeing as the lipid interactions holding together the stratum corneum are lessened/removed in such an environment (80). Once the schistosomes have passed through the upper layers of keratinocytes, they encounter resistance at the stratum spinosus, and proceed to facilitate the killing of nearby cells while also degrading the connections (largely composed of cadherins) between such cells. This process, as well as the subsequent degradation of host structural components, may be facilitated by both mechanical movement as well as factors that degrade cell–cell adhesions. Possible sources for such factors include the head gland, sub-tegumental cell bodies, and acetabular glands (73). While each of these sources may have roles in facilitating penetration, the most likely suspect remains the acetabular glands, cells featuring long duct-like cytoplasmic extensions leading to the anterior of the parasite that are packed with proteases (81). Acetabular gland secretions are produced during migration through the collagen rich basement membrane of the skin and have been confirmed to be secreting up to three days post infection. The cells of the acetabular gland atrophy between 48–72 h post infection, and are not thought to be a key factor in penetrating into host venules, a process which is thought to be mediate by the parasite’s head gland (75).The acetabular glands are, however, necessary for percutaneous infection using mechanically transformed cercariae. Thus, it is likely that these glands remain the predominant method by which human schistosomes facilitate entry through the skin (73, 80, 82–84).

Investigations into the proteolytic factors responsible for skin penetration have revealed of a wide variety of serine proteases as a means by which to degrade host structural components. *S. mansoni*, which has been studied extensively in this area, produces numerous serine proteases, which are expelled from its acetabular glands during penetration (**Figure 2A**). Among these proteases are a 28/30 kDa protease capable of cleaving casein, gelatin, C3, C3b laminin, fibronectin, keratin, and collagens type IV and VIII; a 47 kDa protease capable of cleaving gelatin, casein, collagen type VI, and elastin; and a 60 kDa protease capable of cleaving casein and gelatin (85–87). Inhibition of these serine proteases using serine protease inhibitors reduces the likelihood of successful penetration into human skin (88). Among these serine proteases, the 28/30 kDa variant that is referred to as *Sm*CE (*Schistosoma mansoni* Cercarial Elastase) is the most crucial, as it composes roughly 36% of the total volume of acetabular gland contents (89). Inhibition of *Sm*CE using the protease inhibitor succinyl-alanyl-alanyl-prolyl-phenylalanine chloromethyl ketone (AAPF-CMK) is capable of reducing cercarial penetration by up to 80% (90). Highlighting the importance of *Sm*CE is its persistent presence throughout the intra-mammalian portion of the *S. mansoni* life cycle. A membrane bound version of the protein is found in cercariae, lung stage schistosomulae, and adult worms (91). While *S. haematobium* possess a protease like *Sm*CE, it was long held that *S. japonicum* lacked any serine protease production whatsoever during initial penetration events because *Sm*CE antibodies failed to react with *S. japonicum* cercarial extracts (92). More recent proteomic analysis of the host/parasite molecular interface during *S. japonicum* penetration into mouse skin revealed a single *S. japonicum* cercarial elastase (*Sj*CE2b) made in cercaria and localized to the acetabular glands, although levels of this protein pale in comparison to what is found in *S. mansoni* and *S. haematobium* (93).

Given the relative difference in serine protease abundance between varying species of human schistosomes, other factors must participate in the cercarial penetration event. Cathepsins have emerged as an alternative facilitator of penetration (**Figure 2A**). *S. japonicum* cercaria, despite lower protein output during transformation to schistosomulae than *S. mansoni*, exhibit up to 40-fold more cathepsin-B-like activity than their *S. mansoni* counterparts (92). Additionally, proteomic analysis of the proteases found in *S. japonicum* cercaria and schistosomulae have identified five different cathepsins and only one serine protease (93). Given that *S. japonicum* is considered to be a more ancestral species of schistosome than *S. mansoni* or *S. haematobium*, it is possible that these cathepsins are an evolutionarily older mechanism of facilitating penetration into a wider variety of hosts. Seeing as *S. japonicum* has been observed to elicit more of a swollen red bump at the site of penetration, it is possible that the expanded use of serine proteases in *S. mansoni* in particular could have resulted from an evolutionary attempt to move away from Cathepsins as a penetration facilitating molecule given their immunostimulatory properties in humans (92). *S. mansoni* still produces cathepsins, two of which (Cathepsin L1 and Cathepsin B) are present in the post acetabular glands of the parasite. Given the function of post acetabular glands in producing mucous like substances to facilitate attachment to host skin, it is possible that these cysteine proteases may have a role in overcoming the skin as an immune barrier. Alternatively, the involvement of cathepsin activity in the adult schistosome gut suggests that the presence of cathepsins in cercaria also has the potential to serve as nothing more than evidence of the development of factors necessary for digestion in subsequent life cycle stages (94).

Cercaria, as well as the skin stage schistosomula into which they develop, must also contend with a variety of different immune cells in the skin. The immune response commences early during penetration in the epidermis, with HLA-DR+ cells, likely Langerhans cells, having been shown to aggregate at the sight of infection within 48 h, while keratinocytes respond to penetration by the release of the proinflammatory cytokines IL-1*α* and IL-1β (77, 95). Additionally, fluorescent imaging has revealed that neutrophils, macrophages, and dendritic cells are all capable of internalizing the products released by cercariae upon penetration (96). Eosinophils and neutrophils can kill developing schistosomula with the aid of complement components and immunoglobulins *in vitro*. Thus, one can assert that humans possess a more than adequate array of schistosomicidal immune cells in the skin that could kill the developing larvae in the absence of immunomodulatory factors (65).

Several candidate immune modulators produced by skin staged schistosomula have been identified (**Figure 2A**). However, only a few of which have been functionally characterized to date. Chief among these modulators is an anti-inflammatory protein termed *Sm*16/*Sm*SLP/*Sm*SPO-1. This 16.8 kDa protein composes roughly 3–4% of the protein secreted from cercariae during 0–3 h post infection, suggesting a role in parasite survival (89). It has been demonstrated that *Sm*16 can alter cytokine profiles. It downregulates Il-1*α* production in keratinocytes, lowers ICAM-1 expression in endothelial cells, prevents LPS induced neutrophil movement into the dermis, and reduces LPS mediated IL-6, TNF-α, and IL-1β production. In mice, it lessens the capacity of mouse bone marrow derived macrophages from producing Il-12p40, IL-10, and IFN-*γ*-induced $\text{N}{\text{O}}_{2}^{-}$ production, while also slowing antigen processing by phagocytic cells (97–100). The *Sm*16 counterpart in *S. japonicum*, termed *Sj*16, has also been shown to possess immunomodulatory properties, including a reduction in macrophage maturation, while also modulating cytokine production in thioglycolate-induced peritoneal mouse cells by upregulating IL-10 and IL-1RA, while downregulating MIP-2, IL-1β and IL-12p35 (101). Interestingly, *Sj*16 is also capable of increasing the abundance of CD4^+^CD25^+^ Foxp3^+^ regulatory T cells, thereby suggesting that it not only has the capacity to downregulate inflammatory responses, but may also contribute to the development of a regulatory response (102). Originally, the roughly 30% similarity that *Sm*16 features with human stathmin led researchers to hypothesize that *Sm*16 functioned as a microtubule destabilizing protein. This suggestion has been disproven, and it has been shown that *Sm*16 actually assembles as a 9 unit lipid bilayer-associated oligomer that is capable of altering both TLR4 and TLR3 signaling (99, 103). Despite this plethora of functions, immunization of mice against *Sm*16, as well as infections with *Sm*16 RNAi knockdown parasites reveals that elevated humoral and cellular immunity to *Sm*16 do not confer protective immunity n mice, with worm burden and egg laying remaining unchanged (104). Given that natural infections with *S. mansoni* (in humans) and *S. japonicum* (in rabbits) fail to elicit strong antibody mediated responses, *Sm*16, long considered a possible vaccine candidate, does not merit investigation as such (101, 105).

Other, perhaps less well known/examined immunomodulators are also features of schistosome infections in the human skin (**Figure 2A**). Some, such as the 23 kDa *S. mansoni*-derived apoptosis inducing factor, seemingly exert their immunomodulatory properties by direct targeting of T cells. Schistosome E/S fractions containing a 23 kDa protein have been shown to specifically target T lymphocytes for apoptosis, a process thought to be mediated by causing an upregulation of both the Fas Ligand and Fas receptor on CD3+ cells (106). This destruction of T lymphocytes during initial penetration may be partially to blame for the inability of immunized mouse lymphocytes to recognize the E/S products of invading parasites, as a proper T cell mediated response would be severely hampered (107). Other molecules may act by instead modulating the cytokine environment in which the parasite finds itself. *S. mansoni* can produce prostaglandin E2 (PGE_2_), while also producing an E/S product of less than 30 kDa in size that can upregulate the production PGE_2_ and IL-10 from human keratinocytes. This seems to be of significant importance to the kinetics of infection, as IL-10 deficient mice are able to slow schistosomula travel through the skin and into the lungs (108). Both the 23 kDa and 30 kDa immunomodulatory factors were identified *via* fractionation of schistosome E/S products, with the exact molecular identity having not yet been ascertained. The use of Prostaglandins is not limited to PGE_2_, however, as PGD_2_ produced by the parasite inhibits the migration of epidermal Langerhan cells to nearby lymph nodes (109). Given that production of PGD_2_ by *S. mansoni* has been demonstrated to require a 28 kD Glutathione S-transferase, an interest in exploiting such a factor as a possible vaccine candidate was explored during the early 1990s. Unfortunately, recent phase 3 clinical trials of the *S. haematobium* derived r*Sh*28GST (Bilhvax) vaccine proved ineffective in granting significant immunity (110–113). Finally, a family of venom allergen like proteins was identified as possible immune modulators. The *S. mansoni* genome was demonstrated to feature 29 of these proteins, which are defined by the presence of a Sperm-coating protein/Tpx-1/Ag5/PR-1/Sc7 (SCP/TAPS) domain (114). *Sm*VALS 4, *Sm*VAL 10, and *Sm*VAL 18 were identified as being present in the E/S components from cercaria and compose roughly 3% of the normalized proteins found therein using mass spectrometry. The presence of *Sm*VAL24 in the acetabular glands has been observed *via* whole-mount *in situ* hybridization (89, 115). To date, only two of these *Sm*VAL proteins have been functionally characterized. SmVAL4 possesses lipid and cholesterol binding capacity, although how this might result in the modulation of the host immune response has not yet been examined (116). *Sm*VAL18, on the other hand, has been shown to bind plasminogen, and help facilitate its cleavage into plasmin, which plays a role in the degradation of complement components, extracellular matrix proteins, and fibrinolysis. Thus, *Sm*VAL18 could conceivably help the parasite migrate through the skin and avoid blood clotting during penetration into a venule (117). In *S. japonicum*, only one VAL, *Sj*-VAL-1 has been examined and has been shown to localize to the penetration and head glands of cercariae, suggesting a possible role in migration into host venules (118).

In addition to those immune mediators that have been characterized, numerous other parasite-derived factors hypothesized to be involved in overcoming the immune response in the skin have been identified by genomic, transcriptomics and proteomic approaches (**Figure 2A**). One interesting possible series of targets are metalloproteases. In *S. mansoni*, genomic data suggests the presence of 114 metalloproteases, 35 of which were found to have differential transcription through different live cycle stages (114, 119). Of note was the presence of seven invadolysins, five of which were upregulated preferentially in the germ ball stage of development, and two of which were most upregulated in the cercaria. Given that one invadolysin (Smp_090100.1) constitutes 12.8% of the protein secreted during the first 3 h of infection, while another (Smp_135530) was shown to be a key determinant of infection status in the intermediate host, investigations into the role of such proteases is merited in the context of mammalian infection (55, 89). This is supported by recent findings examining proteins expressed during *S. japonicum* penetration, which suggest the expression of six different invadolysins, five of which continued to be expressed by schistosomula after successful migration through the skin (93). Another potential immunomodulator yet to be functionally characterized is *Sm*KK7, a protein bearing significant homology to K+ channel blockers in scorpion venom, which could foreseeably function in inhibiting the activation of surrounding lymphocytes (89).

Despite the diverse and plentiful existence of these factors secreted by invading cercaria/skin stage schistosomula, schistosomes do not merely rely on the release of immunomodulatory molecules into the skin meant to ward off effector immune cells. The parasite must also deal with the potential of being opsonized by complement components and immunoglobulins and has therefore evolved both surface-associated factors and the use of secreted products to combat host innate and adaptive responses. This process begins during the first 3 h of invasion, wherein the schistosomula rapidly seeks to shed the proteins and carbohydrates composing its glycocalyx (**Figure 2A**). While the glycocalyx serves an important role in mediating survival under the high osmotic pressures seen during the free living aquatic cercarial stage of life, once in the skin, it is a potent target for both the classical and alternative pathways for the complement system (120). While the process of losing the glycocalyx is facilitated in part by mechanical movement throughout the epidermis, the close association of *Sm*CE has been suggested as a possible aid during this process.

While the glycocalyx is shed, schistosomulae also undergo a complex reorganization of their outer membrane in which they go from a trilaminate state into a heptalaminate state that persists into adulthood (121). This newly formed heptalaminate membrane then begins to display several surface-bound factors geared towards disabling complement and immunoglobulin-based attacks. One such molecule is paramyosin, which has been shown in association with both schistosomula and adult worms. Paramyosin has been shown to bind complement components C1, C8, and C9, thereby inhibiting polymerization and deposition of the membrane attack complex on schistosomulae exposed to human serum (122). The membrane has also been shown to feature a receptor capable of binding to the Fc fragment of human IgG (but not IgE, IgA, and IgM), while also binding to the *β*_2_-microglublin found in the human major histocompatibility complex (123). Such binding could conceivably function in masking the schistosomula with host proteins, but the orientation of binding also suggests it is a mechanism of rendering IgG unable to signal to surrounding effector cells. With the Fc portion of IgG bound to the schistosome, the antibody-dependent cytotoxicity employed by cells such as macrophages, neutrophils, and eosinophils cannot occur due to the inability of IgG to bind the Fc receptor on the surface of such cells. Immunoglobulins in the immediate area of the infection appear to be targeted by serine proteases secreted from the schistosomula, as surface bound IgG is cleaved into Fc and soluble Fab fragments in a manner inhibited by the serine protease inhibitor phenylmethylsulfonyl fluoride (PMSF) (124). Additionally, soluble IgE is cleaved by a schistosomula derived serine protease likely to be *Sm*CE, as a specific inhibitor of this factor inhibits IgE cleavage (125).

### Immunosuppression by Schistosomula in the Lungs

After successfully reaching either a venule or lymphatic vessel, schistosomes begin their migration through the bloodstream towards the lungs, where they will mature for a few days prior to continuing to the liver. While some immune mediators used in the skin continue to be used in the lungs (*Sm*16, *Sj*16, venom allergen like proteins, surface paramyosin), exposure of the larvae to lung epithelial cells elicits new immunomodulatory mechanisms (126). This is particularly crucial given the observation that vaccination with radiated parasites results in protective immunity that largely results in parasite killing in the lungs, suggesting that immunosuppression in the lungs is key to survival and establishment in the host (127). Although the entirety of how this resistance is mediated is poorly understood, it appears that several E/S products of lung-stage schistosomula elicit a strong Th1 type immune response that requires cooperation with Th17 and Th2 factors in order to facilitate larval death (128, 129).

To date, the factors produced by lung stage schistosomes remain poorly examined due to difficulty of isolation, prior focus on the skin stage larvae as potential vaccine targets, and a lack of standardization of the transformation and culture conditions during *in vitro* culturing of lung-stage larvae. Despite this, some studies have gone on to perform both transcriptomic analysis of lung stage larvae, as well as proteomic screens, and have identified factors thought to target host leukocytes (130, 131). One protein found to be transcriptionally upregulated was a venom allergen like protein, suggesting the presence of this protein family in yet another life cycle stage of *S. mansoni* (130). Another potential target identified by both a microarray analysis and transcriptomics screen in *S. mansoni* was a protein simply termed Antigen 5 which bears homology to an antigen from *Echinococcus granulosis* hydatid cysts. This antigen is thought to be immunomodulatory, although a particular mechanism has yet to be described (130–132).

When mechanisms designed to hamper the activation and engagement of effector cells fail, schistosomes must be able to withstand to withstand the cytotoxic effects of reactive oxygen species and reactive nitrogen species in order to survive. In fact, immune responses that effectively generate large amounts of these reactive species have been shown to correlate with larval death (128). Schistosomes switch from relying on aerobic metabolism to anaerobic metabolism one during their first two weeks in their definitive host, which is hypothesized to be at least partially responsible for their gain in resistance to nitric oxide mediated killing (133, 134). This is significant given the observation that nitric oxide is a significant determinant of infection success *in vivo*, with nitric oxide synthase knockout *Rattus norvegicus* showing a significant decrease in resistance to *S. japonicum* infection (135). In order to combat H_2_O_2_, however, schistosomes appear to utilize surface-associated peroxiredoxin-1as a scavenger molecule, as evidenced by the fact that RNAi mediated knockdown of this molecule renders *S. japonicum* susceptible to H_2_O_2_ (136).

Finally, schistosomula must also overcome a significant barrier during their trip throughout the vasculature, to the lungs, as well as their adults lives thereafter: human blood. Although leukocytes are correctly pointed to as the main sources of immunity in whole human blood, it is important to recognize that blood coagulation and platelet deposition surrounding pathogens is also a key mechanism by which humans fight off infection. Schistosomes have evolved mechanisms to avoid clot formation in their immediate vicinity. In both *S. japonicum* and *S. mansoni* surface bound enolases are employed as plasminogen binding proteins that increase the amount of active plasmin in close proximity to the parasite. Active plasmin surrounding the parasite results in an increase in fibrinolytic activity (137, 138). Although these enolases have been shown to facilitate this process both *in vivo* and *in vitro*, it would appear as though the plasmin activity facilitated by all three of the major human schistosomes does not rely solely on enolases, as up to 10 plasminogen binding proteins have been identified *via* western blotting techniques. RNAi-mediated KD of enolases does not significantly inhibit schistosomula associated plasmin activity, suggesting other as of yet unidentified factors are also involved in the inhibition of clot formation (137).

### Adults

Adult schistosomes incorporate many different strategies to avoid and overcome the host immune response (**Figure 2C**). The first of these strategies is the capacity of adult schistosomes to incorporate host antigens on their surface in an attempt to hide themselves from host immune factors. This was first suggested by the work of Smithers et al. in which adult worms were transplanted into a host of a different species. After these transplants, the adult worms would usually survive, unless the recipient host had been vaccinated with antigens from the donor host species prior to the infection, in which case most worms would die. This strongly suggested incorporation of host molecules onto the surface of the worm (139). This work would become a staple of parasitology classes and helped lay the foundation for future work into schistosome host antigen absorption. Subsequent work has demonstrated that this incorporation of host products is not a random process, nor a result of the generalized “stickiness” of the glycocalyx. It is, rather, due to the presence of receptors for various host antigens (**Figure 2C**). Receptors for the Fc portion of immunoglobulins, one of which has been shown to be a paramyosin as well as a *β*_2_-microglobulin in a MCH class I receptor, and a C3 receptor explains the presence of these molecules on the surface of the worm (123, 140, 141). Although the binding of IgG *via* its Fc region renders it unable to bind the Fc receptor on circulating effector cells, the exact orientation/function of complement component C3 binding is not known (**Figure 2C**). Adult schistosomes are also capable of incorporate host CD44 into the outer portion of their tegument, especially on the tips of the adult spines (142). To date, an immunological function of CD44 being used by the worm remains to be identified.

Another immune evasion method is molecular mimicry, which is employed by adult schistosomes *via* the production of molecules bearing significant homology to host signaling molecules. Schistosomes have been shown to produce levels of adrenocorticotropic hormones, which, when processed by human polymorphonuclear leukocytes into alpha- melanotropin, results in the subsequent inactivation of these cells, and could potentially decrease Th1 cell activation (143, 144). Additionally, schistosomes have the capacity to generate substances bearing similarities to morphine and codeine, which had the effect of decreasing leukocyte activation (144, 145).

While the exploitation and mimicry of host molecules greatly aid the worm during infection, they are also capable of employing novel self-made mechanisms of interfering with the human immune response (**Figure 2D**). The production of immunomodulatory elements in adults occurs early in their arrival to the hepatic portal system or venous plexus. In *S. japonicum*, unique factors are shown to be produced as early as day 14 post infection. Identified among the E/S proteins from 14-day old *S. japonicum* are a cystatin and an integrin. The cystatin is hypothesized to induce the polarization of alternatively activated macrophages, which are characteristic of a Th2 type responses that are less toxic to larvae than Th1 type inflammatory response. The integrin is hypothesized to mediate T cell migration, which might alter cytokine production in the milieu surrounding the larval parasite, lessening the immune challenges it may face in the lungs (146). While these cell targeting factors have yet to be functionally characterized, at least one *S. japonicum* Thioredoxin peroxidase (*Sj*TPx) has. It is a protein released from 14 day old schistosomula that has been shown to reduce the presence of MHCII and CD86 on LPS stimulated RAW macrophages, thereby implicating it in downregulating of antigen presentation and subsequent T cell activation (146).

Many immunomodulatory factors are found in the tegument, the multi-layered outer surface of the parasite which is capable of quick self-renewal after damage, a process mediated by a population of somatic stem cells with a propensity to differentiate into tegument associated cells (147).The factors within the tegument have been extensively examined (148, 149). In *S. japonicum*, one such factor, *S. japonicum* tetraspanning orphan receptor (*Sj*TOR), proves to be yet another component in fighting complement mediated death, and has been shown to bind complement component C2, while preventing complement mediated cell lysis (150). The tegument is also home to the *S. mansoni* Kunitz type protease inhibitor (*Sm*KI-1), a potent serine protease inhibitor with similarities to the Kunitz type protease inhibitors found in the gut of *Fasciola hepatica* (151). In *S. mansoni*, it is present both in the tegument of adult worms, as well as their E/S products (**Figure 2D**) (152). This particular inhibitor was shown to be crucial for parasite survival within mice, as siRNA KD of *Sm*KI-1 resulted in increased killing *via* neutrophil elastase, a molecule the inhibitor was shown to target *in vitro* (153). *Sm*KI-1 is accompanied in the tegument by *Sm*200, a 200 kDa protein used for diagnostic purposes, but whose only characterized function to date is the causation of an increased abundance in IL-10 secretion from surrounding cells (154). In *S. japonicum*, another protease inhibitor, this one of the cysteine variety, has been shown to inhibit the lysosomal cysteine protease of dendritic cells, while also reducing their antigen processing/presentation on MHC class II (155). Clearly, these two protease inhibitors are evolved mechanisms meant to prevent cell mediated killing should the parasites attempts at avoiding cell recognition/contact fail (155). Lastly, thought-provoking work into the mechanisms by which adult schistosomes release immunomodulatory factors into the host revealed that they are not restricted to proteins shed as part of the membrane, but that they also employ exosome-like vesicles that drive classical activation of macrophages (156–158). Earlier examinations of the surface adult surface demonstrated the release of portions of the tegument, but did not make note of observing such small, exosome like vesicles (121, 159, 160).

Another significant barrier to overcome that adult worms must face is survival in the host bloodstream. Given the long lifespans of adult schistosomes, in addition to their relatively small size (approximately 1cm in length), constant exposure to blood clotting factors put them at risk of immobility and subsequent death. While the presence of the enolases seen in schistosomulae have been confirmed in adults, a bevy of other possible anti-coagulation mechanisms have been identified in adult worms. This is perhaps most clearly demonstrated by the fact that addition of mated pairs of adult worms to murine blood significantly reduces coagulation, while at 7 weeks post infection with *S. mansoni*, mouse blood features clots that break down more easily (161). While this general observation of anticoagulation is interesting, the past few decades have allowed for a more refined understanding of the exact molecules present at the surface of the parasite that allow for such activity (**Figure 2B**). Among the specific anticoagulants discovered are the kininogen cleaving serine protease Sk1, the *α*- and *γ*-thrombin binding/inhibiting membrane bound antigen *Sm*22.6, the ATP-diphosphohydrolase enzyme *Sm*ATPDase1 that cleaves the platelet activator ADP, the more recently characterized tegumental calpains S, Calp1 and *Sm*Calp2 that cleave host fibrinogen, and finally, the secreted serine protease *Sm*SP2 that activates parts of the fibrinolytic system and promotes vasodilation (162–165). The discovery of these specific factors clearly demonstrate that schistosomes have evolved a large variety of methods by which to ensure their free movement and survival within host vasculature (**Figure 2B**) (166). While attacks on the parasite outer surface by blood borne factors are well characterized, less is known about how the parasite survives the host immune mediators that it internalizes during feeding on host erythrocytes. Of note is the observation that one of the several proteases the schistosome gut produces for digestion of RBCs, cathepsin D, is also capable of cleaving IgG and complement component C3, although it is not known if this functions as an immune evasion technique for combatting gut permeabilization or is rather just part of digesting these factors as possible nutrient sources (167). The contents of the adult gut are not limited to proteases, seeing as a recent study demonstrated that a micro-exon gene (MEG) protein MEG-14 interacts with and sequesters the pro-inflammatory human calgranulin S100A9, suggesting a possible role in immune downregulation (168, 169). Perhaps one of the more remarkable things about schistosomes in the blood is their capacity to utilize ingested blood components to modulate the surrounding immune response. This is made evident by the observation that hemozoin, the biproduct of erythrocyte digestion composed of heme molecules, is regurgitated by the worm, taken up by macrophages, and downregulates the Th2 types response seen during egg laying (170).

### Eggs

The eggs laid by schistosomes are the primary cause of pathology associated with human schistosomiasis. This has resulted in significant amounts of research going into understanding the immunological milieu surrounding these eggs. While many aberrantly migrating eggs become lodged in the liver, they typically seek to exit the host *via* the intestines/bladder. The process by which the eggs exit the host have been reviewed extensively; therefore here we seek to highlight the specific factors excreted by the eggs to modulate/downplay the host response during the migration through the host intestines and bladder (171, 172). Schistosome eggs are composed of a thick protein shell, a cell derived envelope responsible for the secretion of various factors, and the developing miracidium (173, 174). Adult female schistosomes are estimated to lay roughly 300 eggs per day during their peak reproductive ages, and while many of these eggs will aberrantly migrate to the liver where they reside until death, successful eggs will begin their migration through the intestine towards the intestinal lumen (175). This process is accompanied by the formation of a granuloma surrounding the eggs. While schistosome egg antigens have been shown to be capable of inducing apoptosis of host cells, the parasite appears to harness the host immune response in order to facilitate egg movement towards the lumen. This is made evident by the fact that egg laying coincides with the appearance of a Th2 type response in the host, and a similar response can by induced *via* the direct injection of eggs into the anterior mesenteric veins of mice (176). In mice, a balance between host Th1 and Th2 responses to schistosome eggs is necessary, seeing as deficiencies in either result in accelerated pathology and subsequent death. That having been said, the observation that IL-4 and Il-13 negative mice rarely pass eggs in their feces suggests that a Th2 type granuloma response is imperative for the egg to reliably exit the intestinal wall successfully (177, 178). To date, two specific molecules have been identified that have been shown to drastically skew the T cell response in granulomas towards a Th2 type response. The first of these is a major glycoprotein known as alpha-1 (α-1), also referred to as the Il-4 inducing principle of *S. mansoni* eggs (IPSE). IPSE is recognized as an antigen by basophils, subsequently resulting in an increased amount of IL-4 production and an alteration of the T helper cell response (**Figure 2E**) (179). Of note is the observation that this molecule is also capable of upregulating Il-10 production in B cells, thereby increasing the proliferation of regulatory T cells, a process that may be more important in dealing with granulomas in the liver (180). Intriguingly, *α*-1 is not the only Th2 inducing glycoprotein secreted by schistosome eggs, as another glycoprotein, Omega1 (Ω-1), a T2 ribonuclease, has shown to be the primary method by which the eggs induce a Th2 response (181). Ω -1 does not affect IL-4 release from basophils like its counterpart, *α*-1, but rather it targets dendritic cell (DC) functions (**Figure 2E**) (182). In 2012, it was shown that Ω-1’s glycosylation patterns allow it to be internalized by DCs *via* binding to the mannose receptor, while ribonuclease activity results in lowered protein synthesis *via* the cleavage of host rRNA and mRNA (183). The significance of this molecule in driving the immune response surrounding schistosome eggs was recently demonstrated in the first ever published occurrence of CRISPR-mediated knockout of a *S. mansoni* gene, which resulted in a decrease in Th2 cytokine production from macrophage/T cell co cultures, while also decreasing the volume of murine pulmonary granulomas (184). This skewing of the immune response allows for granulomas composed of alternatively activated macrophages, dendritic cells, lymphocytes, basophils, and eosinophils. Each of these cell types plays different roles in helping to facilitate movement through the intestinal *via* the degradation of host tissue. This degradation is thought to be aided at least in part by *Sm*Enolase and *Sm*Calp1, whose presence in the eggs is thought to aid in fibrinolysis (137, 163). Additionally the egg is thought to facilitate its own survival by producing *Sm*KI-1 as a method of surviving neutrophil elastase mediate death, while also producing a chemokine binding protein (*Sm*CKBP) that reduces inflammation and inflammatory cell recruitment *via* the binding of CXCL8 and CCL3 (**Figure 2E**) (185). These immune modulating and immune evading tactics allow for the egg to migrate through the host intestine/bladder, so that they may eventually be excreted in order to commence their life cycle anew.

## Conclusion

Schistosomes have evolved an indirect life cycle featuring both an intermediate gastropod host in which they undergo asexual replication and a mammalian definitive host, including humans, in which adult worms inhabiting the bloodstream undergo sexual reproduction. This complex life cycle has led schistosomes to develop a bevy of mechanisms to avoid being killed by the immune system of either the snail or human hosts. In snails, a mixture of molecular mimicry and E/S products from the developing larvae are utilized to target host hemocytes and prevent their movement, engagement, and killing of the parasite. In humans, various proteases are used to enter the host, after which each life cycle stage produces numerous factors meant for the specific targeting of particular cell types relevant to survival at each stage of development within the host. Given recent advancements in praziquantel administration efforts correlating with a decrease in estimated schistosome infections worldwide, hope exists for the eventual elimination of this deadly and debilitating disease (186). That having been said, an estimated 200 million people are still infected with schistosomiasis, highlighting the need for alternative therapeutics, as well as the possible development of a vaccine. Research into understanding the mechanisms employed by the parasite to survive in both hosts remains crucial in better understanding infection outcomes. The progression in this field from basic observational research all the way to targeted gene deletions suggests a bright future for research into schistosome immune evasion strategies.

## Author Contributions

All authors listed have made a substantial, direct, and intellectual contribution to the work and approved it for publication.

## Funding

This work was supported by the Natural Sciences and Engineering Council of Canada #2018-05209 and 2018-522661 (PCH) and an Alberta Innovates Health Solutions Graduate Studentship - 201810623-RA (JRH).

## Conflict of Interest

The authors declare that the research was conducted in the absence of any commercial or financial relationships that could be construed as a potential conflict of interest.
